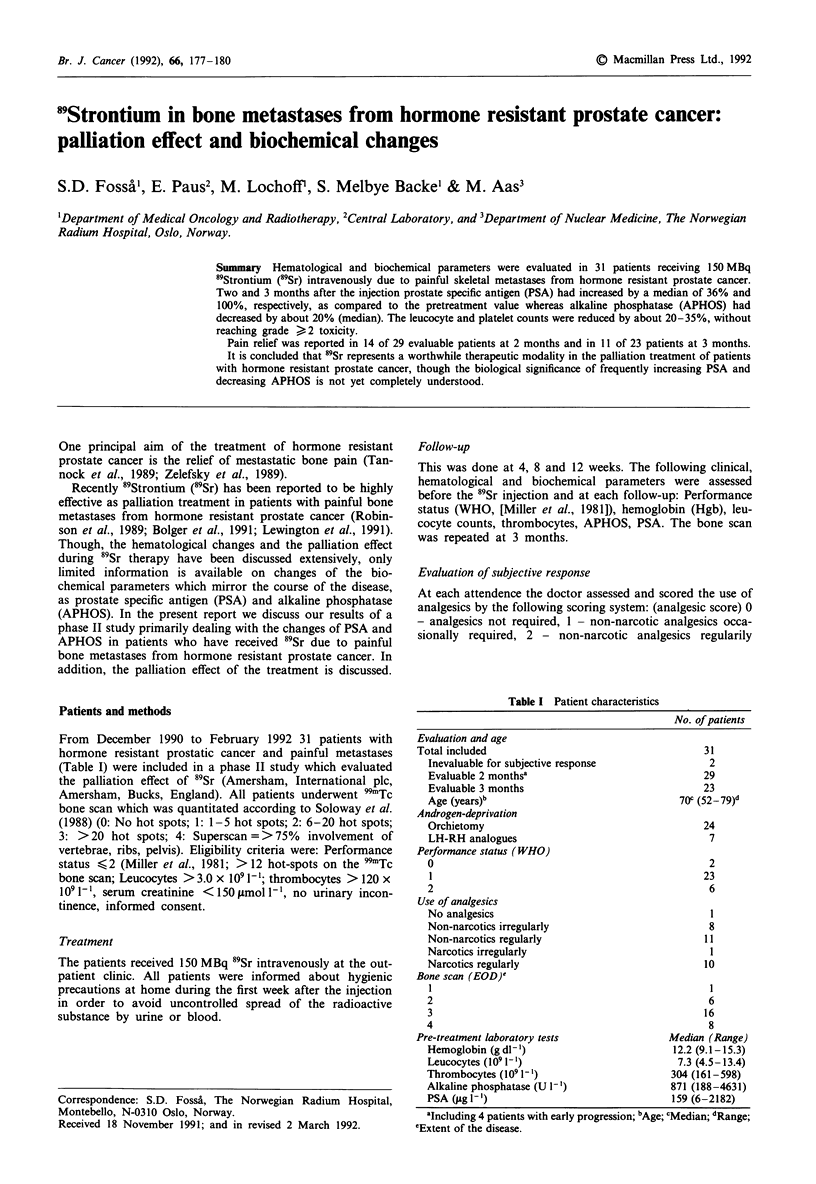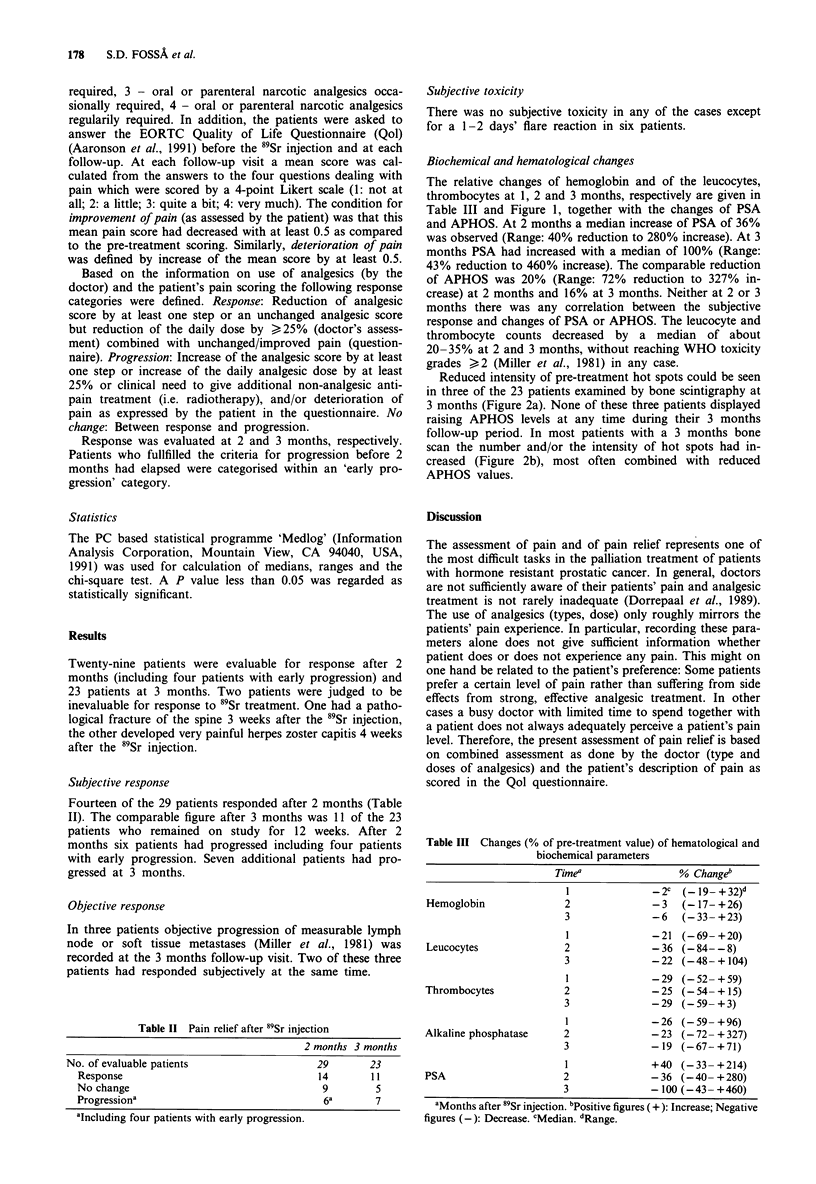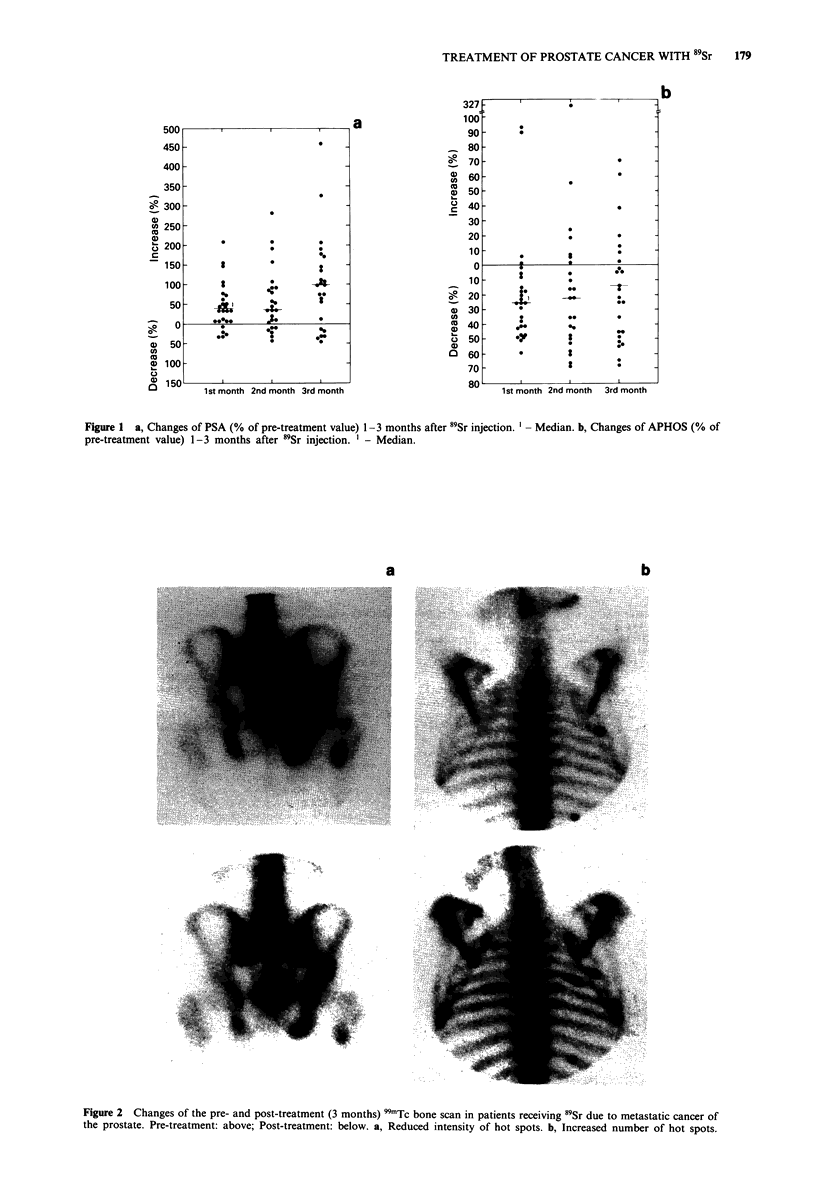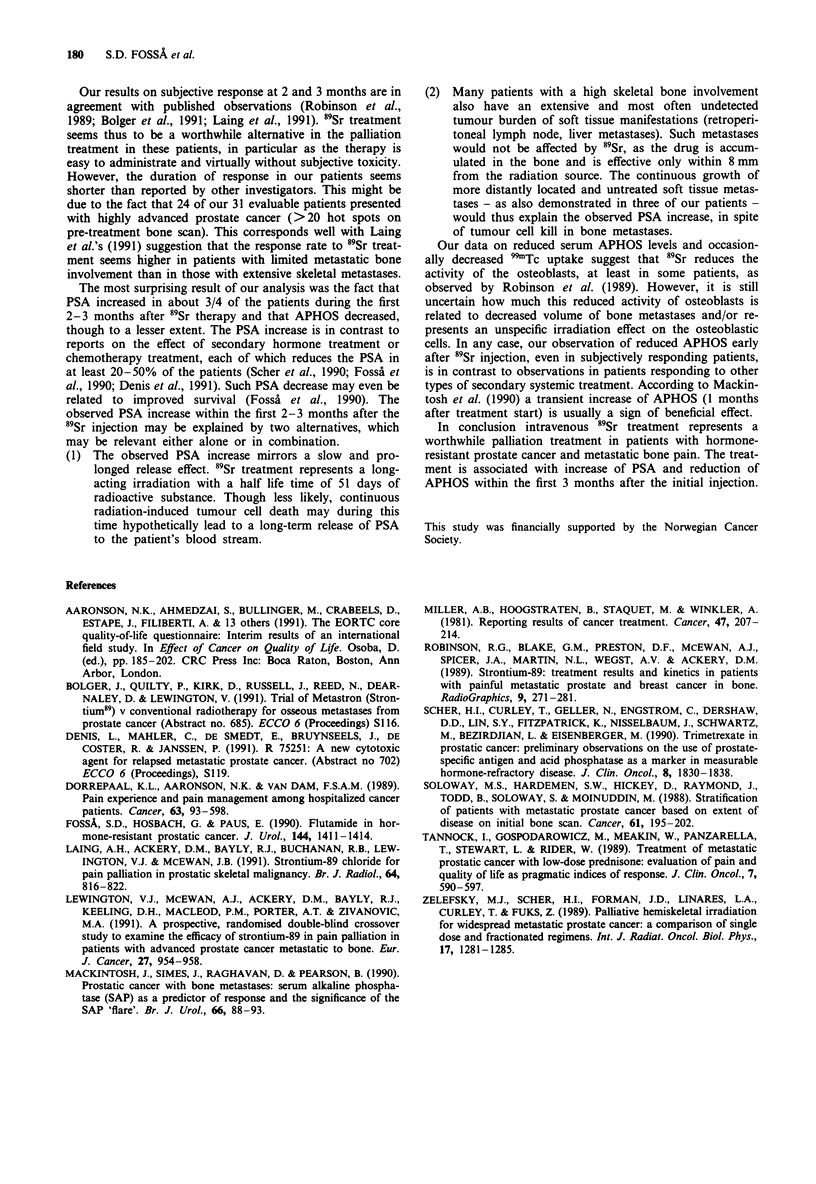# 89Strontium in bone metastases from hormone resistant prostate cancer: palliation effect and biochemical changes.

**DOI:** 10.1038/bjc.1992.238

**Published:** 1992-07

**Authors:** S. D. Fosså, E. Paus, M. Lochoff, S. M. Backe, M. Aas

**Affiliations:** Department of Medical Oncology and Radiotherapy, Norwegian Radium Hospital, Oslo.

## Abstract

**Images:**


Br. J. Cancer (1992), 66, 177-180 © Macmillan Press Ltd., 1992

### $^{89}$Strontium in bone metastases from hormone resistant prostate cancer: palliation effect and biochemical changes

S.D. Fosså[1], E. Paus[2], M. Lochoff[1], S. Melbye Backe[1] & M. Aas[3]

[1]*Department of Medical Oncology and Radiotherapy,* [2]*Central Laboratory, and* [3]*Department of Nuclear Medicine, The Norwegian Radium Hospital, Oslo, Norway.*

**Summary** Hematological and biochemical parameters were evaluated in 31 patients receiving 150 MBq $^{89}$Strontium ($^{89}$Sr) intravenously due to painful skeletal metastases from hormone resistant prostate cancer. Two and 3 months after the injection prostate specific antigen (PSA) had increased by a median of 36% and 100%, respectively, as compared to the pretreatment value whereas alkaline phosphatase (APHOS) had decreased by about 20% (median). The leucocyte and platelet counts were reduced by about 20-35%, without reaching grade $\geqslant 2$ toxicity.

Pain relief was reported in 14 of 29 evaluable patients at 2 months and in 11 of 23 patients at 3 months. It is concluded that $^{89}$Sr represents a worthwhile therapeutic modality in the palliation treatment of patients with hormone resistant prostate cancer, though the biological significance of frequently increasing PSA and decreasing APHOS is not yet completely understood.

One principal aim of the treatment of hormone resistant prostate cancer is the relief of mestastatic bone pain (Tannock *et al.*, 1989; Zelefsky *et al.*, 1989).

Recently $^{89}$Strontium ($^{89}$Sr) has been reported to be highly effective as palliation treatment in patients with painful bone metastases from hormone resistant prostate cancer (Robinson *et al.*, 1989; Bolger *et al.*, 1991; Lewington *et al.*, 1991). Though, the hematological changes and the palliation effect during $^{89}$Sr therapy have been discussed extensively, only limited information is available on changes of the biochemical parameters which mirror the course of the disease, as prostate specific antigen (PSA) and alkaline phosphatase (APHOS). In the present report we discuss our results of a phase II study primarily dealing with the changes of PSA and APHOS in patients who have received $^{89}$Sr due to painful bone metastases from hormone resistant prostate cancer. In addition, the palliation effect of the treatment is discussed.

### Patients and methods

From December 1990 to February 1992 31 patients with hormone resistant prostatic cancer and painful metastases (Table I) were included in a phase II study which evaluated the palliation effect of $^{89}$Sr (Amersham, International plc, Amersham, Bucks, England). All patients underwent $^{99m}$Tc bone scan which was quantitated according to Soloway *et al.* (1988) (0: No hot spots; 1: 1-5 hot spots; 2: 6-20 hot spots; 3: >20 hot spots; 4: Superscan = >75% involvement of vertebrae, ribs, pelvis). Eligibility criteria were: Performance status $\leqslant 2$ (Miller *et al.*, 1981; >12 hot-spots on the $^{99m}$Tc bone scan; Leucocytes $> 3.0 \times 10^9 l^{-1}$; thrombocytes $> 120 \times 10^9 l^{-1}$, serum creatinine $< 150 \mu mol l^{-1}$, no urinary incontinence, informed consent.

### *Treatment*

The patients received 150 MBq $^{89}$Sr intravenously at the outpatient clinic. All patients were informed about hygienic precautions at home during the first week after the injection in order to avoid uncontrolled spread of the radioactive substance by urine or blood.

### *Follow-up*

This was done at 4, 8 and 12 weeks. The following clinical, hematological and biochemical parameters were assessed before the $^{89}$Sr injection and at each follow-up: Performance status (WHO, [Miller *et al.*, 1981]), hemoglobin (Hgb), leucocyte counts, thrombocytes, APHOS, PSA. The bone scan was repeated at 3 months.

### *Evaluation of subjective response*

At each attendence the doctor assessed and scored the use of analgesics by the following scoring system: (analgesic score) 0 - analgesics not required, 1 - non-narcotic analgesics occasionally required, 2 - non-narcotic analgesics regularly

**Table I** Patient characteristics

| | *No. of patients* |
|---|---|
| *Evaluation and age* | |
| Total included | 31 |
| Inevaluable for subjective response | 2 |
| Evaluable 2 months[a] | 29 |
| Evaluable 3 months | 23 |
| Age (years)[b] | 70[c] (52-79)[d] |
| *Androgen-deprivation* | |
| Orchietomy | 24 |
| LH-RH analogues | 7 |
| *Performance status (WHO)* | |
| 0 | 2 |
| 1 | 23 |
| 2 | 6 |
| *Use of analgesics* | |
| No analgesics | 1 |
| Non-narcotics irregularly | 8 |
| Non-narcotics regularly | 11 |
| Narcotics irregularly | 1 |
| Narcotics regularly | 10 |
| *Bone scan (EOD)[e]* | |
| 1 | 1 |
| 2 | 6 |
| 3 | 16 |
| 4 | 8 |
| *Pre-treatment laboratory tests* | *Median (Range)* |
| Hemoglobin ($g dl^{-1}$) | 12.2 (9.1-15.3) |
| Leucocytes ($10^9 l^{-1}$) | 7.3 (4.5-13.4) |
| Thrombocytes ($10^9 l^{-1}$) | 304 (161-598) |
| Alkaline phosphatase ($U l^{-1}$) | 871 (188-4631) |
| PSA ($\mu g l^{-1}$) | 159 (6-2182) |

[a]Including 4 patients with early progression; [b]Age; [c]Median; [d]Range; [e]Extent of the disease.

Correspondence: S.D. Fosså, The Norwegian Radium Hospital, Montebello, N-0310 Oslo, Norway.
Received 18 November 1991; and in revised 2 March 1992.

required, 3 – oral or parenteral narcotic analgesics occasionally required, 4 – oral or parenteral narcotic analgesics regularly required. In addition, the patients were asked to answer the EORTC Quality of Life Questionnaire (Qol) (Aaronson *et al.*, 1991) before the $^{89}Sr$ injection and at each follow-up. At each follow-up visit a mean score was calculated from the answers to the four questions dealing with pain which were scored by a 4-point Likert scale (1: not at all; 2: a little; 3: quite a bit; 4: very much). The condition for *improvement of pain* (as assessed by the patient) was that this mean pain score had decreased with at least 0.5 as compared to the pre-treatment scoring. Similarly, *deterioration of pain* was defined by increase of the mean score by at least 0.5.

Based on the information on use of analgesics (by the doctor) and the patient's pain scoring the following response categories were defined. *Response*: Reduction of analgesic score by at least one step or an unchanged analgesic score but reduction of the daily dose by ⩾25% (doctor's assessment) combined with unchanged/improved pain (questionnaire). *Progression*: Increase of the analgesic score by at least one step or increase of the daily analgesic dose by at least 25% or clinical need to give additional non-analgesic anti-pain treatment (i.e. radiotherapy), and/or deterioration of pain as expressed by the patient in the questionnaire. *No change*: Between response and progression.

Response was evaluated at 2 and 3 months, respectively. Patients who fullfilled the criteria for progression before 2 months had elapsed were categorised within an 'early progression' category.

### *Statistics*

The PC based statistical programme 'Medlog' (Information Analysis Corporation, Mountain View, CA 94040, USA, 1991) was used for calculation of medians, ranges and the chi-square test. A $P$ value less than 0.05 was regarded as statistically significant.

### Results

Twenty-nine patients were evaluable for response after 2 months (including four patients with early progression) and 23 patients at 3 months. Two patients were judged to be inevaluable for response to $^{89}Sr$ treatment. One had a pathological fracture of the spine 3 weeks after the $^{89}Sr$ injection, the other developed very painful herpes zoster capitis 4 weeks after the $^{89}Sr$ injection.

### *Subjective response*

Fourteen of the 29 patients responded after 2 months (Table II). The comparable figure after 3 months was 11 of the 23 patients who remained on study for 12 weeks. After 2 months six patients had progressed including four patients with early progression. Seven additional patients had progressed at 3 months.

### *Objective response*

In three patients objective progression of measurable lymph node or soft tissue metastases (Miller *et al.*, 1981) was recorded at the 3 months follow-up visit. Two of these three patients had responded subjectively at the same time.

**Table II** Pain relief after $^{89}Sr$ injection

| | *2 months* | *3 months* |
|---|---|---|
| No. of evaluable patients | *29* | *23* |
| Response | 14 | 11 |
| No change | 9 | 5 |
| Progression[a] | 6[a] | 7 |

[a]Including four patients with early progression.

### *Subjective toxicity*

There was no subjective toxicity in any of the cases except for a 1–2 days' flare reaction in six patients.

### *Biochemical and hematological changes*

The relative changes of hemoglobin and of the leucocytes, thrombocytes at 1, 2 and 3 months, respectively are given in Table III and Figure 1, together with the changes of PSA and APHOS. At 2 months a median increase of PSA of 36% was observed (Range: 40% reduction to 280% increase). At 3 months PSA had increased with a median of 100% (Range: 43% reduction to 460% increase). The comparable reduction of APHOS was 20% (Range: 72% reduction to 327% increase) at 2 months and 16% at 3 months. Neither at 2 or 3 months there was any correlation between the subjective response and changes of PSA or APHOS. The leucocyte and thrombocyte counts decreased by a median of about 20–35% at 2 and 3 months, without reaching WHO toxicity grades ⩾2 (Miller *et al.*, 1981) in any case.

Reduced intensity of pre-treatment hot spots could be seen in three of the 23 patients examined by bone scintigraphy at 3 months (Figure 2a). None of these three patients displayed raising APHOS levels at any time during their 3 months follow-up period. In most patients with a 3 months bone scan the number and/or the intensity of hot spots had increased (Figure 2b), most often combined with reduced APHOS values.

### Discussion

The assessment of pain and of pain relief represents one of the most difficult tasks in the palliation treatment of patients with hormone resistant prostatic cancer. In general, doctors are not sufficiently aware of their patients' pain and analgesic treatment is not rarely inadequate (Dorrepaal *et al.*, 1989). The use of analgesics (types, dose) only roughly mirrors the patients' pain experience. In particular, recording these parameters alone does not give sufficient information whether patient does or does not experience any pain. This might on one hand be related to the patient's preference: Some patients prefer a certain level of pain rather than suffering from side effects from strong, effective analgesic treatment. In other cases a busy doctor with limited time to spend together with a patient does not always adequately perceive a patient's pain level. Therefore, the present assessment of pain relief is based on combined assessment as done by the doctor (type and doses of analgesics) and the patient's description of pain as scored in the Qol questionnaire.

**Table III** Changes (% of pre-treatment value) of hematological and biochemical parameters

| | *Time*[a] | *% Change*[b] |
|---|---|---|
| | 1 | −2[c] (−19–+32)[d] |
| Hemoglobin | 2 | −3 (−17–+26) |
| | 3 | −6 (−33–+23) |
| | 1 | −21 (−69–+20) |
| Leucocytes | 2 | −36 (−84–−8) |
| | 3 | −22 (−48–+104) |
| | 1 | −29 (−52–+59) |
| Thrombocytes | 2 | −25 (−54–+15) |
| | 3 | −29 (−59–+3) |
| | 1 | −26 (−59–+96) |
| Alkaline phosphatase | 2 | −23 (−72–+327) |
| | 3 | −19 (−67–+71) |
| | 1 | +40 (−33–+214) |
| PSA | 2 | −36 (−40–+280) |
| | 3 | −100 (−43–+460) |

[a]Months after $^{89}Sr$ injection. [b]Positive figures (+): Increase; Negative figures (−): Decrease. [c]Median. [d]Range.

**Figure 1** a, Changes of PSA (% of pre-treatment value) 1–3 months after $^{89}Sr$ injection. [1] – Median. b, Changes of APHOS (% of pre-treatment value) 1–3 months after $^{89}Sr$ injection. [1] – Median.

**Figure 2** Changes of the pre- and post-treatment (3 months) $^{99m}Tc$ bone scan in patients receiving $^{89}Sr$ due to metastatic cancer of the prostate. Pre-treatment: above; Post-treatment: below. a, Reduced intensity of hot spots. b, Increased number of hot spots.

Our results on subjective response at 2 and 3 months are in agreement with published observations (Robinson *et al.*, 1989; Bolger *et al.*, 1991; Laing *et al.*, 1991). $^{89}$Sr treatment seems thus to be a worthwhile alternative in the palliation treatment in these patients, in particular as the therapy is easy to administrate and virtually without subjective toxicity. However, the duration of response in our patients seems shorter than reported by other investigators. This might be due to the fact that 24 of our 31 evaluable patients presented with highly advanced prostate cancer ($>$20 hot spots on pre-treatment bone scan). This corresponds well with Laing *et al.*'s (1991) suggestion that the response rate to $^{89}$Sr treatment seems higher in patients with limited metastatic bone involvement than in those with extensive skeletal metastases.

The most surprising result of our analysis was the fact that PSA increased in about 3/4 of the patients during the first 2–3 months after $^{89}$Sr therapy and that APHOS decreased, though to a lesser extent. The PSA increase is in contrast to reports on the effect of secondary hormone treatment or chemotherapy treatment, each of which reduces the PSA in at least 20–50% of the patients (Scher *et al.*, 1990; Fosså *et al.*, 1990; Denis *et al.*, 1991). Such PSA decrease may even be related to improved survival (Fosså *et al.*, 1990). The observed PSA increase within the first 2–3 months after the $^{89}$Sr injection may be explained by two alternatives, which may be relevant either alone or in combination.

(1) The observed PSA increase mirrors a slow and prolonged release effect. $^{89}$Sr treatment represents a long-acting irradiation with a half life time of 51 days of radioactive substance. Though less likely, continuous radiation-induced tumour cell death may during this time hypothetically lead to a long-term release of PSA to the patient's blood stream.

(2) Many patients with a high skeletal bone involvement also have an extensive and most often undetected tumour burden of soft tissue manifestations (retroperitoneal lymph node, liver metastases). Such metastases would not be affected by $^{89}$Sr, as the drug is accumulated in the bone and is effective only within 8 mm from the radiation source. The continuous growth of more distantly located and untreated soft tissue metastases – as also demonstrated in three of our patients – would thus explain the observed PSA increase, in spite of tumour cell kill in bone metastases.

Our data on reduced serum APHOS levels and occasionally decreased $^{99m}$Tc uptake suggest that $^{89}$Sr reduces the activity of the osteoblasts, at least in some patients, as observed by Robinson *et al.* (1989). However, it is still uncertain how much this reduced activity of osteoblasts is related to decreased volume of bone metastases and/or represents an unspecific irradiation effect on the osteoblastic cells. In any case, our observation of reduced APHOS early after $^{89}$Sr injection, even in subjectively responding patients, is in contrast to observations in patients responding to other types of secondary systemic treatment. According to Mackintosh *et al.* (1990) a transient increase of APHOS (1 months after treatment start) is usually a sign of beneficial effect.

In conclusion intravenous $^{89}$Sr treatment represents a worthwhile palliation treatment in patients with hormone-resistant prostate cancer and metastatic bone pain. The treatment is associated with increase of PSA and reduction of APHOS within the first 3 months after the initial injection.

This study was financially supported by the Norwegian Cancer Society.

### References

AARONSON, N.K., AHMEDZAI, S., BULLINGER, M., CRABEELS, D., ESTAPE, J., FILIBERTI, A. & 13 others (1991). The EORTC core quality-of-life questionnaire: Interim results of an international field study. In *Effect of Cancer on Quality of Life*. Osoba, D. (ed.), pp. 185–202. CRC Press Inc: Boca Raton, Boston, Ann Arbor, London.

BOLGER, J., QUILTY, P., KIRK, D., RUSSELL, J., REED, N., DEARNALEY, D. & LEWINGTON, V. (1991). Trial of Metastron (Strontium[89]) v conventional radiotherapy for osseous metastases from prostate cancer (Abstract no. 685). *ECCO 6* (Proceedings) S116.

DENIS, L., MAHLER, C., DE SMEDT, E., BRUYNSEELS, J., DE COSTER, R. & JANSSEN, P. (1991). R 75251: A new cytotoxic agent for relapsed metastatic prostate cancer. (Abstract no 702) *ECCO 6* (Proceedings), S119.

DORREPAAL, K.L., AARONSON, N.K. & VAN DAM, F.S.A.M. (1989). Pain experience and pain management among hospitalized cancer patients. *Cancer*, **63**, 93–598.

FOSSÅ, S.D., HOSBACH, G. & PAUS, E. (1990). Flutamide in hormone-resistant prostatic cancer. *J. Urol.*, **144**, 1411–1414.

LAING, A.H., ACKERY, D.M., BAYLY, R.J., BUCHANAN, R.B., LEWINGTON, V.J. & MCEWAN, J.B. (1991). Strontium-89 chloride for pain palliation in prostatic skeletal malignancy. *Br. J. Radiol.*, **64**, 816–822.

LEWINGTON, V.J., MCEWAN, A.J., ACKERY, D.M., BAYLY, R.J., KEELING, D.H., MACLEOD, P.M., PORTER, A.T. & ZIVANOVIC, M.A. (1991). A prospective, randomised double-blind crossover study to examine the efficacy of strontium-89 in pain palliation in patients with advanced prostate cancer metastatic to bone. *Eur. J. Cancer*, **27**, 954–958.

MACKINTOSH, J., SIMES, J., RAGHAVAN, D. & PEARSON, B. (1990). Prostatic cancer with bone metastases: serum alkaline phosphatase (SAP) as a predictor of response and the significance of the SAP 'flare'. *Br. J. Urol.*, **66**, 88–93.

MILLER, A.B., HOOGSTRATEN, B., STAQUET, M. & WINKLER, A. (1981). Reporting results of cancer treatment. *Cancer*, **47**, 207–214.

ROBINSON, R.G., BLAKE, G.M., PRESTON, D.F., MCEWAN, A.J., SPICER, J.A., MARTIN, N.L., WEGST, A.V. & ACKERY, D.M. (1989). Strontium-89: treatment results and kinetics in patients with painful metastatic prostate and breast cancer in bone. *RadioGraphics*, **9**, 271–281.

SCHER, H.I., CURLEY, T., GELLER, N., ENGSTROM, C., DERSHAW, D.D., LIN, S.Y., FITZPATRICK, K., NISSELBAUM, J., SCHWARTZ, M., BEZIRDJIAN, L. & EISENBERGER, M. (1990). Trimetrexate in prostatic cancer: preliminary observations on the use of prostate-specific antigen and acid phosphatase as a marker in measurable hormone-refractory disease. *J. Clin. Oncol.*, **8**, 1830–1838.

SOLOWAY, M.S., HARDEMEN, S.W., HICKEY, D., RAYMOND, J., TODD, B., SOLOWAY, S. & MOINUDDIN, M. (1988). Stratification of patients with metastatic prostate cancer based on extent of disease on initial bone scan. *Cancer*, **61**, 195–202.

TANNOCK, I., GOSPODAROWICZ, M., MEAKIN, W., PANZARELLA, T., STEWART, L. & RIDER, W. (1989). Treatment of metastatic prostatic cancer with low-dose prednisone: evaluation of pain and quality of life as pragmatic indices of response. *J. Clin. Oncol.*, **7**, 590–597.

ZELEFSKY, M.J., SCHER, H.I., FORMAN, J.D., LINARES, L.A., CURLEY, T. & FUKS, Z. (1989). Palliative hemiskeletal irradiation for widespread metastatic prostate cancer: a comparison of single dose and fractionated regimens. *Int. J. Radiat. Oncol. Biol. Phys.*, **17**, 1281–1285.